# Variation-Based Distance and Similarity Modeling: A Case Study in World Englishes

**DOI:** 10.3389/frai.2019.00023

**Published:** 2019-11-05

**Authors:** Benedikt Szmrecsanyi, Jason Grafmiller, Laura Rosseel

**Affiliations:** ^1^Department of Linguistics, Faculty of Arts, KU Leuven, Leuven, Belgium; ^2^Department of English Language and Linguistics, University of Birmingham, Birmingham, United Kingdom; ^3^Linguistic and Literary Studies (LIST), Faculty of Arts and Philosophy, Vrije Universiteit Brussel, Brussels, Belgium

**Keywords:** comparative sociolinguistics, VADIS, probabilistic grammar, dialectometry, variationist linguistics

## Abstract

Inspired by work in comparative sociolinguistics and quantitative dialectometry, we sketch a corpus-based method (Variation-Based Distance & Similarity Modeling—VADIS for short) to rigorously quantify the similarity between varieties and dialects as a function of the correspondence of the ways in which language users choose between different ways of saying the same thing. To showcase the potential of the method, we present a case study that investigates three syntactic alternations in some nine international varieties of English. Key findings include that (a) probabilistic grammars are remarkably similar and stable across the varieties under study; (b) in many cases we see a cluster of “native” (a.k.a. Inner Circle) varieties, such as British English, whereas “non-native” (a.k.a. Outer Circle) varieties, such as Indian English, are a more heterogeneous group; and (c) coherence across alternations is less than perfect.

## Introduction

Determining whether different varieties, dialects, or languages for that matter share the same or a similar “grammar” is an important and theoretically significant topic in comparative linguistics. In this paper we present a variationist method (Variation-Based Distance & Similarity Modeling—VADIS for short) to determine such similarity, based on naturalistic corpus and hence production data. VADIS builds bridges between subfields in sociolinguistics and variation studies that should be allied but that are in practice surprisingly disjoint. First, dialectometry (see e.g., Séguy, 1971; Goebl, 1982; Nerbonne et al., 1999) is concerned with aggregate measures of linguistic similarity and distance as a function of geographic space; what is at issue is inter-speaker variation, where language users of dialect A use form X and language users of dialect B use form Y. Second, variationist linguistics (see e.g., Labov, 1969; Gries, 2003; Bresnan et al., 2007) takes an interest in how speakers choose between formally distinct variants to express the same meaning, subject to probabilistic constraints that may be language-internal, stylistic, or language-external in nature; variationist linguistics, then, is in the first place all about intra-speaker variability (or “variability in the linguistic signal within a given language,” in the parlance of van Hout and Muysken, 2016, p. 250), that is, variation between forms that are in principle available to all members of a given speech community. The basic idea behind VADIS is to use the output of variationist modeling as an input to dialectometric analysis, or—in other words—to measure inter-speaker variation by assessing the structure of intra-speaker variability.

Why do we need VADIS? There is, of course, an extensive literature on how to determine the grammatical similarity of varieties and dialects based on dialect atlases or survey data (for example, Spruit et al., 2009; Szmrecsanyi and Kortmann, 2009; Cysouw, 2013). Using naturalistic corpus data to measure the grammatical similarity of varieties is a trickier task. One avenue consists of establishing the text frequencies of forms and constructions in corpora, and to distill geolinguistic patterns from the frequency signal (Szmrecsanyi, 2013; Grieve, 2016). But VADIS digs even deeper than that: what counts is not if and/or how often people use particular constructions, but how they choose between “alternate ways of saying ‘the same' thing” (Labov, 1972, p. 188). VADIS takes advantage of the fact that variationist analysis is good at quantifying the probabilistic grammar(s)—the set of constraints and their probabilistic effects on how people choose between variants of a particular variable^1^—of intra-speaker variation, and essentially defines the similarity between varieties as being proportional to how similar the probabilistic grammars regulating variation are. This is a more thoroughgoing, less “surfacy” method in comparison to the above-mentioned classical similarity-estimation methods: note that two dialects may have the exact same inventory of forms, and (though unlikely) these forms may even occur with the exact same text frequency—but still, the probabilistic conditioning of the forms may vary. VADIS is the only currently available method that will work under such circumstances.

VADIS builds on methods developed in comparative sociolinguistics (e.g., Tagliamonte, 2001), which has been used for decades to evaluate the relatedness of typically a small number of dialects drawing on multivariate evidence of typically a single variation phenomenon: are the same constraints significant across varieties? Do the constraints have similar effect sizes? Is the overall ranking of constraints similar? Unlike classical comparative sociolinguistics, however, VADIS scales up better to the study of a potentially infinite number of varieties based on many variation phenomena.

To showcase the descriptive and theoretical potential of the VADIS method, we analyze by way of a case study similarity patterns and relationships between varieties of English, fueled by a variationist analysis of three syntactic alternations:

1. The genitive alternation (Heller et al., 2017)
  - *the country's economic crisis* (the *s*-genitive)
  - *the economic growth of the country* (the *of*-genitive)
2. The dative alternation (Röthlisberger et al., 2017)
  - *I'd given Heidi my T-Shirt* (the ditransitive dative variant)
  - *I'd given the key to Helen* (the prepositional dative variant)
3. The particle placement alternation (Grafmiller and Szmrecsanyi, 2018)
  - *just cut the tops off* (verb-object-particle order)
  - *cut off the flowers* (verb-particle-object order)

In principle, it is the analyst's decision which alternation(s) to include in the analysis; VADIS does not impose any restrictions, as long as linguistic choice-making can be modeled as a function of clearly defined language-internal and and/or language-external probabilistic constraints. In the case study at hand, the three alternations above were selected as they are all positional alternations subject to similar probabilistic constraints (e.g., constituent weight, constituent animacy, and so on).

The alternations in (1–3) are studied in nine World Englishes (British English, Canadian English, Irish English, New Zealand English, Hong Kong English, Indian English, Jamaican English, Philippine English, and Singapore English), based on materials from the International Corpus of English (ICE) and the Corpus of Global Web-Based English (GloWbE). Relevant observations of the (a) and (b) variants above were annotated for ~10 probabilistic constraints including e.g., the principle of end weight (longer constituents tend follow shorter constituents; see e.g., Wasow and Arnold, 2003) and animacy effects (animate constituents tend to occur early; see e.g., Rosenbach, 2008).

Analysis indicates, among other things, that (a) probabilistic grammars are remarkably similar and stable across the varieties under study; (b) in many cases we see a cluster of “native” (a.k.a. Inner Circle) varieties, such as British English, whereas “non-native” (a.k.a. Outer Circle) varieties, such as Indian English, are a more heterogeneous group; and (c) coherence across alternations is less than perfect.

This paper is structured as follows: Section Data discusses the datasets we investigate. Section Spelling out the Variation-Based Distance & Similarity Modeling (VADIS) Method explains the VADIS method. In sections Quantification via similarity coefficients, Mapping out (dis)similarity relationships between varieties, and Assessing coherence, we present results. Section Discussion and Conclusion offers a discussion and conclusion.

## Data

In this paper, we re-analyze the genitive alternation dataset investigated by Heller (2018), the dative alternation dataset investigated by Röthlisberger (2018), and the particle placement dataset investigated by Grafmiller and Szmrecsanyi (2018) (see examples (1–3) above). The three datasets have been created in the context of the same project, and share the same basic design. With an interest in comparative probabilistic variation analysis, team members tapped into the International Corpus of English^2^ (ICE) (Greenbaum, 1991) and the Corpus of Global Web-based English^3^ (GloWbE) (Davies and Fuchs, 2015) to investigate syntactic variability in the following nine varieties of English:

- British English (henceforth: BrE)
- Canadian English (CanE)
- Irish English (IrE)
- New Zealand English (NZE)
- Jamaican English (JamE)
- Singapore English (SgE)
- Indian English (IndE)
- Hong Kong English (HKE)
- Philippine English (PhlE)

ICE, initiated in 1990, is an ongoing project which was designed to create a set of parallel, balanced corpora representative of language usage across a wide range of (standard) national varieties. Each ICE component contains 500 texts of ~2,000 words each, sampled from 12 spoken and written genres/registers. ICE components included here contain data from the early 1990s, with some also containing data collected as late as the early 2000s. Sampling for each national component is conducted by local teams following a common corpus design and annotation scheme to ensure maximal comparability across the components. GloWbE contains data collected from 1.8 million English language websites—both blogs and general web pages—from 20 different countries (~1.8 billion words in all). To keep the datasets to a manageable size, texts were randomly sampled from each of the nine varieties in GloWbE, totaling 500,000 words per variety.

Areally, we are dealing with a convenience sample, subject to the limits of the availability of corpora. But a deliberate attempt was made to evenly balance what (e.g., Kachru, 1985, 1992) has called “Inner Circle” varieties of English (BrE, IrE, CanE, and NZE) and “Outer Circle” varieties of English (JamE, SgE, IndE, HKE, and PhlE). The distinction between Inner Circle and Outer Circle varieties is roughly equivalent to McArthur (1998) distinction between English as a Native Language (ENL) varieties (about communities “in which the language is spoken and handed down as the mother tongue of the majority of the population”; Schneider, 2011, p. 30), and English as a Second Language (ESL) varieties (about communities “in which English has been strongly rooted for historical reasons and assumes important internal functions (often alongside indigenous languages), e.g., in politics (sometimes as an official or co-official language), education, the media, business life, the legal system, etc.”; Schneider, 2011, p. 30). We know from the literature (see Szmrecsanyi and Röthlisberger, 2019 for discussion) that this is a very important dialect-typological distinction in English linguistics.

The goal was to compile datasets amenable to variationist analysis. That means that in a first step interchangeable genitive, dative, and particle placement variants were defined which could be paraphrased by the competing variant with no semantic change. So, for example, (4a) can be paraphrased by (4b), which is why (4a) is a token that would have been included in the dataset, but (5a) cannot—in any of the varieties we study—be paraphrased by (5b), which is why (5a) is not a token that would have been included in the dataset

(4) a. *the speech of the president*
b. *the president's speech*
(5) a. three *liters of wine*
b.? *wine's three liters*

For reasons of space, we cannot review the definitions of the variable contexts in detail here; the reader is referred to the discussions in Heller (2018); Röthlisberger (2018), and Grafmiller and Szmrecsanyi (2018).

After all interchangeable variants were identified in the materials (dative alternation: *N* = 13,171; genitive alternation: *N* = 13,798; particle placement alternation: *N* = 11,454), each observation was annotated, manually or automatically, for a multitude of known and less-well known constraints on syntactic variation. For example, the principle of end-weight (Behaghel, 1909; Wasow and Arnold, 2003) predicts that in VO languages such as English, “heavy” constituents should follow “lighter” constituents. Thus, team members determined (a) the length of the possessor and possessum phrases in the genitive alternation (prediction: comparatively long possessors should favor the *of*-genitive, because the *of*-genitive places the possessor phrase after the possessum phrase), (b) the length of the recipient and theme phrases in the dative alternation (prediction: comparatively long recipients should favor the prepositional dative, because the prepositional dative places the recipient phrase after the theme phrase), and (c) the length of the direct object in the particle placement alternation (prediction: long direct objects favor verb-particle-object order, which places the direct object after the particle). Again, for reasons of space we cannot discuss the annotation procedure in detail; the reader is referred to Heller (2018); Röthlisberger (2018), and Grafmiller and Szmrecsanyi (2018).

## Spelling out the Variation-Based Distance and Similarity Modeling (VADIS) Method

### Overview

VADIS is designed to measure the (dis)similarity of grammars. Grammar is understood here as a set of probabilistic grammars (a.k.a. “variable grammars” in variationist sociolinguistics parlance) conditioning a set of *N* ≥ 1 alternations or variation phenomena (a.k.a. “variables” in variationist sociolinguistics parlance). A probabilistic grammar specifies the set of constraints (a.k.a. predictors or “conditioning factors” in variationist sociolinguistics parlance) regulating a given alternation.

VADIS builds on methods developed in comparative sociolinguistics (see e.g., Tagliamonte, 2001, 2012, 162–173; Tagliamonte et al., 2016), which is a sub-discipline in variationist sociolinguistics that evaluates the relatedness between varieties and dialects based on how similar the conditioning of variation is in these varieties. Comparative sociolinguists rely on three what they call “lines of evidence” to determine relatedness:

1. Are the same constraints significant across varieties?
2. Do the constraints have the same strength across varieties?
3. Is the constraint hierarchy similar?

Similarity thus assessed is then often interpreted as historical and genetic relatedness. VADIS draws inspiration from this literature and adapts the comparative sociolinguistics method so that it can be applied to datasets sampling (a) more than a couple of dialects or varieties, and (b) more than one variation phenomenon at a time. This is accomplished through more rigorous quantification.

Let us illustrate by coming back to our case study, which covers three syntactic alternations in some nine regional varieties of English. Our point of departure is the view that the dative, genitive, and particle placement alternations are alternations between different forms that have the same meaning. We specifically consider each alternation as coming with its own probabilistic grammar, which regulates how people choose between variants. For example, Bresnan et al. (2007) is a seminal study that calculates regression models that predict how speakers of US American English choose between ditransitive (e.g., *I'd given Heidi my T-Shirt*) and prepositional dative variants (e.g., *I'd given my T-Shirt to Heidi*). According to the formula of model A (Bresnan et al., 2007; **Figure 4**), a non-given theme significantly decreases the odds that speakers will choose a prepositional dative variant by some 67% (*b* = −1.1), while an inanimate recipient significantly *in*creases the odds for a prepositional dative variant by a factor of about 12 (*b* = 2.5). These effects are part of the probabilistic grammar that regulates dative choice in spoken US American English, as sampled in the Switchboard corpus. But what would happen if we fitted a parallel model on data of, say, British English? Would we obtain a different model formula? Would the same constraints be significant? Would they have the same effect size? VADIS is a method to address these questions in a rigorously quantitative fashion. The basic idea behind VADIS is that similarity between varieties is proportional to how similar probabilistic grammars and model formulas are.

### The VADIS Pipeline

Practically speaking, VADIS consists of the following steps:

**Step 1:** define, per alternation, the *p* most important constraints on variation. In the case study we are reporting here, we set *p* = 8^4^ and so include the eight most important predictors (across all varieties) for each alternation^5^. We thus choose, in the case study at hand, to hold the number of constraints constant across alternations for the sake of maximum comparability, but we stress that in principle, the number of constraints do not need to be the same, considering that some alternations would naturally lend themselves to having more constraints than others, depending on the extent of previous research and the complexity of the factors at play. To identify the most important predictors, we fit conditional random forest models across all varieties (i.e., not accounting for variety differences) and created a global variable importance ranking of the predictors; we also consulted the extant literature on the alternations in question. Other ways to define predictor sets are certainly possible, but this task is best left to the VADIS user, not to the method itself. In the case of multi-level categorical predictors, we simplified to binary contrasts whenever possible. The predictor sets thus generated are reported in Table 1. We skip a detailed discussion of individual predictors and instead refer the reader to the publications where the annotation of predictors are discussed in detail.

**Table 1:** Predictor sets used for the analysis.

**Genitive alternation (see Heller et al., 2017)**	**Dative alternation (see Röthlisberger et al., 2017)**	**Particle placement alternation (see Grafmiller and Szmrecsanyi, 2018)**
Possessor animacy (animate vs. inanimate)	Log weight ratio between recipient and theme	Length of the direct object in words
Possessor length in words	Recipient pronominality (pronominal vs. non-pronominal)	Definiteness of the direct object (definite vs. indefinite)
Possessum length in words	Theme complexity (complex vs. simple)	Givenness of the direct object (given vs. new)
Possessor NP expression type (NP vs. NC vs. other)	Theme head frequency	Concreteness of the direct object (concrete vs. non-concrete)
Final sibilancy in possessor (present vs. absent)	Theme pronominality (pronominal vs. non-pronominal)	Thematicity of the direct object
Previous choice (*of* vs. *s* vs. none)	Theme definiteness (definite vs. indefinite)	Directional modifier (present vs. absent)
Semantic relation (prototypical vs. non-prototypical)	Recipient givenness (given vs. new)	Semantics (compositional vs. non-compositional)
Possessor head frequency	Recipient head frequency	Surprisal.P

**Step 2:** Fit a series of mixed-effects logistic regression models, one per variety and alternation. The response variable is variant choice (e.g., *s*-genitive vs. *of*-genitive), and the independent variables are the predictor sets identified in step 1. Note that, following Gelman (2008), all numeric variables in the model should be standardized and categorical variables should be centered. This approach allows direct comparison of the magnitudes of the coefficients in the model. We use mixed-effects models (R function glmer()) with random intercepts for speaker/writer (approximated by corpus file id) and genre. Additional random intercepts were possessor and possessum head for the genitive alternation, verb and theme head for the dative alternation and particle verb and head of the direct object for the particle placement alternation. In previous studies, from which these data were taken, random slopes for a number of predictors were initially tried and evaluated. In most cases, models failed to converge, and in those that were successful, the random slopes were not statistically justified. In our experience, this is quite common with corpus-based grammatical alternation studies, where the individual group levels of the random effects (typically texts and/or lexical items) tend to be sparsely populated. There is also growing evidence that imposing maximal random effects structure where it is not supported can adversely affect results (Bates et al., 2015; Matuschek et al., 2017). Therefore we did not include random slopes for this study. The resulting models are of satisfactory quality: concordance statistic (*C*) values^6^ are consistently greater than 0.88, and VIFs never exceed 2.5.

**Step 3:** Based on the variety-specific regression models, determine cross-variety similarity based on predictor significance^7^. In this step, we define the probabilistic distance between two varieties as being proportional to the extent to which the varieties do *not* overlap with regard to which constraints significantly (in the case study at hand, we set alpha = 0.05^8^) regulate variant choice. To exemplify, consider two hypothetical varieties A and B and five constraints a-e which regulate some variation phenomenon:

**Table:** 

	**Variety A**	**Variety B**
Constraint a	Significant	Significant
Constraint b	Significant	Not significant
Constraint c	Not significant	Significant
Constraint d	Not significant	Not significant
Constraint e	Significant	Significant

Variety A and B agree on the significance of three constraints (a, d, e), and disagree with regard to two constraints. The distance between the two varieties is thus two out of five squared Euclidean distance points. Scaling this to an interval between 0 (no disagreement whatsoever) and 1 (maximal disagreement) yields, in the fictitious example at hand, a distance value of 2/5 = 0.4 and a corresponding similarity value of 3/5 = 0.6.

**Step 4:** Based on the variety-specific regression models, determine cross-variety distance and similarity based on the magnitude of effects. To define the similarity between the varieties, this step compares the extent to which the effect sizes of the constraints in the various regression models are similar (inspired by the procedure sketched in Heller, 2018). This is done by calculating a distance matrix based on the model estimates (using Euclidean distance), whether or not they are significant^9^.This is illustrated with a toy example in Tables 2, 3. Table 2 shows the model estimates of five constraints for three varieties. The Euclidean distances between these varieties, based on the estimates from Table 2, are presented in Table 3. The next step for this line of evidence is to calculate the mean distance per variety, i.e., the average of the pairwise distances between the varieties (cf. Table 4). To scale the distances to an interval between 0 and 1, we can ask the following question: what is the maximal distance between the varieties under study? We define this maximal distance here as the distance between two hypothetical varieties whose constraints have exactly the opposite effects. Such cases of complete constraint “flipping”, i.e., a systematic reversal in the direction of *every constraint's effect* between two varieties, are very unlikely to happen in real world contexts. We set the absolute size of all the constraints to a reasonable value (±1) to create two (hypothetical) varieties that are about as different from one another as we could realistically expect two related varieties to be. For the toy case involving 5 constraints in Table 2, the maximum distance is calculated to be 4.47. We divide the observed distances by this value to give normalized distances within a range of 0 to 1. For the similarity scores we subtract these scaled distances from 1 to give us a score where larger values represent greater average similarity (cf. Table 4). Averaging over the similarities in our toy example gives a similarity coefficient of 0.42.

**Table 2:** Model estimates for three fictitious varieties A, B, and C.

	**Variety A**	**Variety B**	**Variety C**
Constraint	−2.10	−1.50	1.20
Constraint	−1.30	−1.60	−1.20
Constraint	0.75	−0.05	0.63
Constraint	0.69	0.80	2.20
Constraint	−0.92	−1.0	−0.79

**Table 3:** Distance matrix for fictitious varieties A, B, and C (Euclidean distance).

	**Variety A**	**Variety B**	**Variety C**
Variety A	0		
Variety B	1.05	0	
Variety C	3.63	3.15	0

**Table 4:** Mean distances and mean similarities per variety.

**Variety**	**Mean distance**	**Mean distance (scaled)**	**Mean similarity**
Variety B	2.10	0.47	0.53
Variety A	2.34	0.52	0.48
Variety C	3.39	0.76	0.24
Mean	2.61	0.58	0.42

**Step 5:** Fit a series of conditional random forest models, one per variety and alternation. To independently estimate the relative importance of the constraints, we use permutation-based variable importance rankings derived from conditional random forests (CRFs; Strobl et al., 2009). Like regression models, random forests are a supervised learning method that aims to predict an outcome from a set of predictor values, however, this is where the similarities end. Random forests are a decision tree-based ensemble method which offers various advantages over regression models. Random forests are more reliable with unbalanced data, and offer methods for assessing the conditional importance of individual predictors in CRFs. Additionally, cross-validation is built into the method, resulting in greater accuracy and more reliable importance measures. For these reasons we believe CRFs offer a valuable independent assessment of the relationship between the alternations and their constraints. For calculating the CRFs and variable importances we use the cforest() and varimpAUC() functions in R's party package^10^. The response variable and independent variables in the models are the same as for the regression models in step 2 (though inputs are not standardized for the CRFs)^11^.

**Step 6:** Based on the variety-specific conditional random forest models, determine cross-variety distance and similarity based on the importance rankings of the predictors. In this last step, we measure the probabilistic distance between two varieties simply as the Spearman rank correlation between those varieties' respective variable importance rankings^12^. For example, consider the three hypothetical varieties A, B, and C with the constraint rankings below:

**Table:** 

	**Variety A**	**Variety B**	**Variety C**
Constraint a	1	1	2
Constraint b	2	3	4
Constraint c	3	2	3
Constraint d	4	4	1
Constraint e	5	5	5

Varieties A and B show the greatest degree of similarity, with a correlation of ρ = 0.9, while varieties A and C are least similar, with a correlation of ρ = 0.3. Variety B is slightly more similar to variety C than variety A is (ρ = 0.4), but it is far more similar to A than to C. We can arrange these pairwise correlations in a table like so:

**Table:** 

	**Variety A**	**Variety B**	**Variety C**
Variety A	1	0.9	0.3
Variety B	0.9	1	0.4
variety C	0.3	0.4	1

From the workflow described above, it is clear that the case study reported in this paper (analyzing the similarity of nine varieties based on three alternations, including various subsets of the data) generated hundreds of regression and CRF models. Hence, it is not possible to report a comprehensive overview of model quality measures for the case studies. Instead, we restrict ourselves reporting the C values for the regression models based on all available data in Table 5 below.

**Table 5:** *C* values for glmer models and CRFs based on all available data.

	**Dative alternation**		**Genitive alternation**		**Particle placement alternation**	
	**Glmer model**	**CRF**	**Glmer model**	**CRF**	**Glmer model**	**CRF**
BrE	0.95	0.95	0.91	0.93	0.89	0.91
CanE	0.96	0.95	0.92	0.93	0.91	0.91
HKE	0.95	0.94	0.92	0.92	0.90	0.93
IndE	0.96	0.96	0.92	0.93	0.88	0.93
IrE	0.95	0.95	0.90	0.92	0.89	0.91
JamE	0.97	0.96	0.92	0.93	0.88	0.93
NZE	0.95	0.94	0.91	0.92	0.91	0.92
PhlE	0.96	0.97	0.90	0.91	0.89	0.94
SgE	0.95	0.95	0.91	0.92	0.91	0.93

An R package (under development) which performs all the above calculation is available at https://github.com/jasongraf1/VADIS. The analysis scripts we used to conduct our case study are available at https://osf.io/3gfqn/, along with the genitive alternation and dative alternation datasets (the particle placement dataset is built into the R package mentioned above).

### About Concept Validity and Reliability

Given the novelty and complexity of the VADIS methodology, some evaluation of the method's validity and reliability is warranted. Preliminary work suggests that the similarity coefficients do indeed accurately and consistently capture relative degrees of similarity among varieties. In a study using a series of simulated datasets, designed with varying degrees of similarity, Heller (2018, p. 199–204) showed that the similarity coefficients derived from models fit to these datasets correlated inversely with the degree of variability built into the data simulation. The more variable the datasets were designed to be when they were created, the lower the similarity coefficients were for all three lines of evidence. In a second study, Röthlisberger (2018, p. 175; 215–216) used a bootstrapping procedure to assess the reliability of the similarity coefficients for each line of evidence across 1,000 bootstrap samples of her datives dataset. She found a high degree of consistency for all three lines of evidence with the second line (coefficient strength) being the most consistent and the third line (constraint ranking) being the least consistent. Finally, we assessed the validity of methods for visualizing similarities (visualization and mapping is discussed in section Mapping Out (dis)Similarity Relationships Between Varieties) via a second simulation study in which artificial datasets were constructed to vary in specific ways and then subjected to VADIS analysis. Results of the visualizations were exactly as predicted, e.g., datasets that were designed to have opposite constraint effects were maximally distinguished, while datasets designed to have nearly identical constraint effects clustered tightly together. In all, we conclude that the procedure is quite robust.

## Quantification via Similarity Coefficients

One way in which VADIS can address the issue of variation-based similarities consists of calculating what we will call here similarity coefficients. The idea is to quantify the similarity between varieties by coefficients which range between 0 and 1, where 0 indicates total dissimilarity and 1 indicates total similarity. Similarity coefficients are calculated as follows: for every variation phenomenon under study, we obtain *n* × (*n*−1)/2 unique pairwise similarity values for each line of analysis (steps 3, 4, and 6), where *n* is the number of varieties under analysis. For example, if we study, say, the dative alternation in 9 varieties, then we obtain 9 × 8/2 = 36 pairwise similarity values for each of the three lines of evidence. Subsequently, we calculate one mean similarity coefficient per line of evidence by simply taking the arithmetic mean of all pairwise similarity values. In the case study at hand with 9 varieties of English, this means that each of the similarity coefficients averages over 36 pairwise similarity values.

Table 6 displays similarity coefficients across lines of evidence and alternations, based on all available data and including all nine regional varieties of English under study. The coefficients range between 0.46 (2nd line, particle placement alternation) and 0.83 (3rd line, genitive alternation). The last row displays mean similarity coefficients per alternation across lines of evidence. So the mean similarity coefficient for the genitive alternation is 0.74; for the dative alternation it is 0.64; and for the particle placement alternation it is 0.68. In other words, the genitive alternation is most stable across varieties, and the dative alternation is least stable; the particle placement alternation takes the middle road. As far as the three different lines of evidence are concerned, we note that the 1st line (significance) and the 3rd line (constraint ranking) yield on average similarly sized coefficients; 2nd line measurements (effect strength) are substantially lower in the case of the genitive and dative alternations, though not in the particle placement alternation.

**Table 6:** Similarity coefficients across lines of evidence and alternations.

	**Genitive alternation**	**Dative alternation**	**Particle alternation**	
1st line (significance)	0.81	0.68	0.73	
2nd line (effect strength)	0.60	0.46	0.69	
3rd line (ranking)	0.83	0.78	0.62	
mean	0.74	0.64	0.68	
				Γ = 0.69

*Input dataset: all available data. Coefficients range between 0 (total dissimilarity) and 1 (total similarity)*.

The value in the bottom row of the rightmost column of Table 6 is what we would like to call the core grammar score (Γ): it is the mean similarity coefficient across all alternations subject to study and thus abstracts away from particular alternations. In the case study at hand (3 syntactic alternations × 9 varieties of English; all available data), we obtain a core grammar score of Γ = 0.69. Relying on customary schemes for interpreting (correlation) coefficients (e.g., De Vaus, 2002, p. 272), we thus see “substantial to very strong” similarities between the varieties under study.

The foregoing analysis is based on all available data. What would happen if we restricted attention to particular subsets of the data? Table 7 reports core grammar scores Γ for a number of sub-datasets, along with hierarchies of stability as far as individual alternations are concerned. When VADIS is run on particular sub-datasets (as opposed to the full dataset), then, core grammar scores tend to be higher, thanks to the fact the sub-datasets in question are by definition more homogeneous (spoken only, Inner Circle only, etc.) The largest core grammar score is obtained when attention is restricted to Inner Circle varieties (Γ = 0.80), indicating that these varieties are particularly homogeneous and similar to each other. Outer Circle varieties are substantially less homogeneous, with a core grammar score of Γ = 0.73. As to the difference that medium makes, written varieties are somewhat more homogeneous (Γ = 0.75) than spoken varieties (Γ = 0.72). Turning to differences between alternations, we have seen before that when we investigate all available data, the hierarchy of stability is genitives > particles > datives (meaning that the way language users choose between genitive variants is most similar across varieties, while dative choices are least similar). The genitive alternation turns out to be most stable also when we restrict attention to various sub-datasets, with the exception of the spoken sub-dataset, where the genitive alternation is actually the least stable one. This is primarily due to a very low similarity coefficient (0.37) for the 3rd line of evidence in spoken materials, meaning that the rankings of constraints on genitive variation are rather dissimilar across varieties.

**Table 7:** Core grammar scores (*Γ*) and hierarchies of stability for subsets of the data.

	**Core grammar score (Γ)**	**Hierarchy of stability**
All available data (Table 6)	Γ = 0.69	Genitives > particles > datives
Spoken data only (ICE-s)	Γ = 0.72	Datives > particles > genitives
Written data only (ICE-w and GloWbE)	Γ = 0.75	Genitives > datives > particles
Inner Circle varieties only (BrE, IrE, CanE, NZE)	Γ = 0.80	Genitives > particles > datives
Outer Circle varieties only (HKE, SgE, IndE, JamE, PhlE)	Γ = 0.73	Genitives > datives > particles

## Mapping out (dis)similarity Relationships Between Varieties

We have seen in the preceding section how VADIS yields similarity coefficients to precisely quantify the (dis)similarity between regionally specific probabilistic grammars. In the case study we have investigated, we have seen that the similarity coefficients tend toward the similarity pole—for example, the core grammar score calculated on the basis of all available data came out at Γ = 0.69 (again, on a scale between 0—indicating maximal dissimilarity—and 1—indicating maximal similarity). So there is clearly more similarity than dissimilarity, but crucially core grammar scores are mean values, and (dis)similarities are not necessarily evenly spread across the network of varieties under study. In this section we will demonstrate how VADIS can be used to visually depict (dis)similarity relationships between varieties.

The aim, then, is not to calculate *mean* similarity coefficients, but to arrange *pairwise* similarity coefficients in so-called distance matrices. Distance matrices are the customary input in classical dialectometry (Séguy, 1971; Goebl, 1982; Nerbonne et al., 1999; Szmrecsanyi, 2013) and work essentially like distance tables in road atlases, which specify geographic distances between locations. Let us illustrate drawing on our case study: for each alternation and each of the three lines of evidence, we create one distance matrix. We are exploring *n* = 9 varieties of English, which yields *n* × (*n*−1)/2 = 9 × 8/2 = 36 unique variety pairings. To each pairing, we assign the relevant inverse similarity coefficient (1—similarity coefficient), thus converting similarity coefficients into *dis*similarity values^13^.

**Figure 1:**
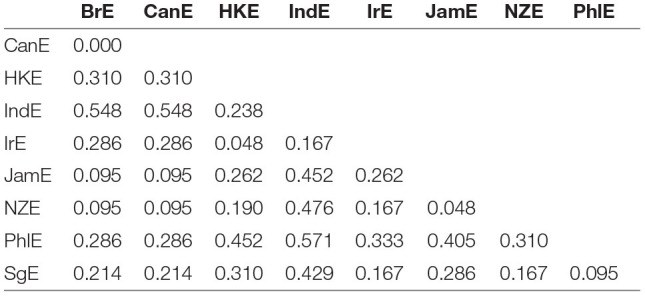
VADIS distance matrix for the 3rd line of evidence in the particle placement alternation (all data included). Scores range between 0 (maximal similarity) and 1 (maximal dissimilarity).

Figure 1 exemplifies by displaying the distance matrix for the 3rd line of evidence (constraint ranking) in the particle placement alternation. All distances are scaled between 0 (no distance) and 1 (maximal distance). Consider now e.g., the pairing between BrE and NZE, which is associated with a comparatively small distance value of 0.095. This is another way of saying that the similarity coefficient associated with this pairing is 1–0.095 = 0.905. In plain English, BrE, and NZE are very similar in terms of the constraint ranking in the particle placement alternation. By contrast, the distance between BrE and IndE is 0.548, which is considerably larger.

**Figure 2:**
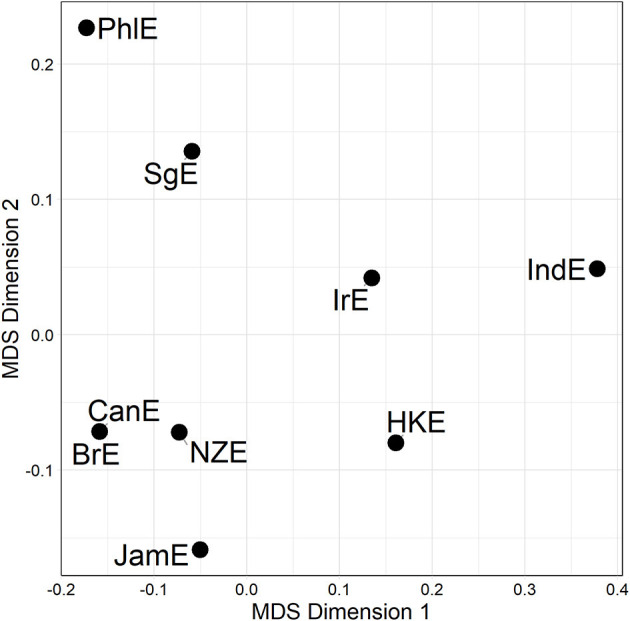
MDS representation of 3rd line distances for the particle placement alternation (see Figure 1). Distances between data points in plot is proportional to probabilistic grammar distances between varieties.

Distance matrices are informative but somewhat hard to process via eye balling. But there are a number of techniques in the dialectometric toolbox to visualize distance matrices. One of these is Multidimensional Scaling (MDS) (see e.g., Kruskal and Wish, 1978), which reduces a higher-dimensional distance matrix to a lower-dimensional representation which is more amenable to visualization^14^. The task before us here is to scale down the *n*−1 dimensional distance matrix (in which each of the nine varieties under study is characterized by its distance to the other eight varieties in the matrix) to a two-dimensional representation. Per alternation, we are initially dealing with three separate distance matrices (one per line of evidence), which could in principle be plotted separately. For example, Figure 2 is a MDS representation of the distance matrix shown in Figure 1. Proximity in the plot is proportional to linguistic similarity. BrE and NZE are close in the plot, while BrE and IndE are fairly distant—which is of course in line with the numerical values in Figure 1.

**Figure 3:**
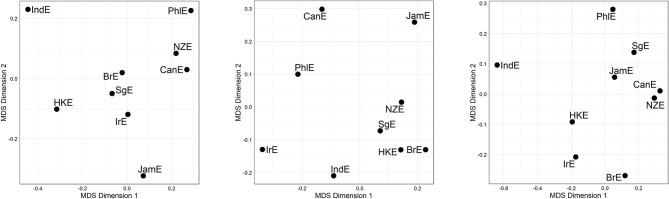
MDS representation of compromise distances per alternation. **(Left)** genitive alternation. **(Middle)** dative alternation. **(Right)** particle placement alternation. Distances between data points in plot is proportional to probabilistic grammar distances between varieties.

Let us now abstract away from individual lines of evidence by fusing the three line-specific distance matrices, thus arriving at line-merged but alternation-specific distance matrices^15^. Figure 3 displays the corresponding MDS plots. Cursory inspection of the plots reveals substantial differences between alternations (we will come back to this issue in the next section), but also similarities—for instance, across all three alternations, IndE and PhlE are at the periphery.

**Figure 4:**
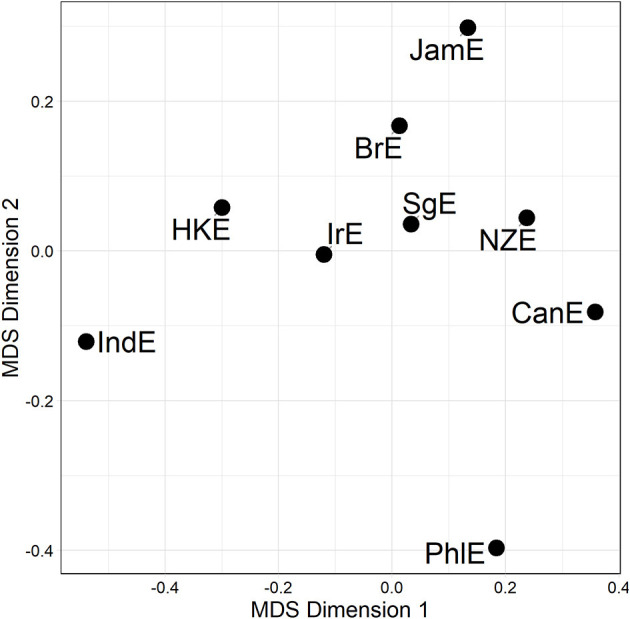
MDS representation of the Γ-matrix (a single compromise distance matrix merged across all lines and alternations). Distances between data points in plot is proportional to probabilistic grammar distances between varieties.

We may now take a further aggregation step for the sake of raising the analysis of (dis)similarity relationships to an even higher level of generalization. This we can accomplish by fusing the three alternation-specific-distance matrices (visualized in Figure 3) into a single compromise distance matrix merged across all lines and alternations, or Γ-matrix for short. An MDS visualization of this Γ-matrix is shown in Figure 4. In the plot, all Inner Circle varieties are clustered in the top right-hand quadrant, with SgE—which according to the literature is an Outer Circle variety in the process of becoming an Inner Circle variety (Leimgruber, 2013, p. 122)—forming part of that cluster. IndE and PhlE are outliers. Supplementary inspection of silhouette widths in hierarchical agglomerative cluster analysis (Levshina, 2015, p. 312) indicates that the distance matrix underlying Figure 4 lacks substantial cluster structure.

## Assessing Coherence

Using the VADIS method means taking a lot of measurements. This section will discuss the extent to which these various measurements overlap with each other. We begin by exploring coherence between the three lines of evidence (constraint significance, constraint strength, and constraint ranking). The question is if large differences between any two varieties according to one particular line of evidence predict large differences between the same two varieties also according to the other lines of evidence. To exemplify, let us re-consider the distance matrix in Figure 1, which is about distances between varieties according to the 3rd line of evidence (constraint ranking) in the particle placement alternation. Figure 1 showed that according to the 3rd line of evidence, BrE and NZE are comparatively close linguistically, while BrE and IndE are comparatively distant. The question is if BrE and NZE will also turn out as close, and BrE and IndE as distant, according to the other lines of evidence.

We measure overlap between distance matrices using the Mantel test (Levshina, 2015, p. 348–349), which, based on permutation, yields correlation coefficients that range between 0 (no overlap) and 1 (total overlap)^16^. Table 8 displays the results. Observe, first, that the dative alternation is the odd one out in that none of the lines overlap with each other in this alternation. Second, the genitive alternation and the particle placement alternation are similar in that they both show moderate but significant overlap between the first line of evidence (constraint significance) and the second line of evidence (constraint strength), as well as substantial overlap between the second line of evidence and the third line of evidence (constraint ranking). We do not see significant overlap anywhere between the first line of evidence and the third line of evidence.

**Table 8:** Mantel correlation coefficients between distance matrices, based on all available data.

	**Genitive alternation**	**Dative alternation**	**Particle alternation**
Overlap 1st line/2nd line	***r*** **=** **0.41 (*****p*** **=** **0.03)**	*r* = 0.12 (*p* = 0.34)	***r*** **=** **0.36 (*****p*** **=** **0.05)**
Overlap 1st line/3rd line	*r* = 0.07 (*p* = 0.36)	*r* = −0.01 (*p* = 0.50)	*r* = 0.25 (*p* = 0.13)
Overlap 2nd line/3rd line	***r*** **=** **0.47 (*****p*** **=** **0.03)**	*r* = −0.15 (*p* = 0.77)	***r*** **=** **0.68 (*****p*** **=** **0.00)**

*Significant coefficients are bolded*.

A related issue concerns the overlap, or coherence, between different alternations. We are concretely asking the following question: if, according to alternation A, two varieties are close in terms of how people choose between different ways of saying the same thing, will the two varieties also turn out to be close when the analysis is based on alternations B and C? Again, we turn to calculating Mantel coefficients between the relevant distance matrices (Table 9).

**Table 9:** Mantel correlation coefficients between fused distance matrices (combining all lines of evidence and based on all available data).

Overlap genitive alternation/dative alternation	*r* = 0.05 (*p* = 0.41)
Overlap genitive alternation/particle alternation	***r* = 0.52 (*p* = 0.01)**
Overlap dative alternation/particle alternation	*r* = 0.11 (*p* = 0.31)

*Significant coefficients are bolded*.

**Figure 5:**
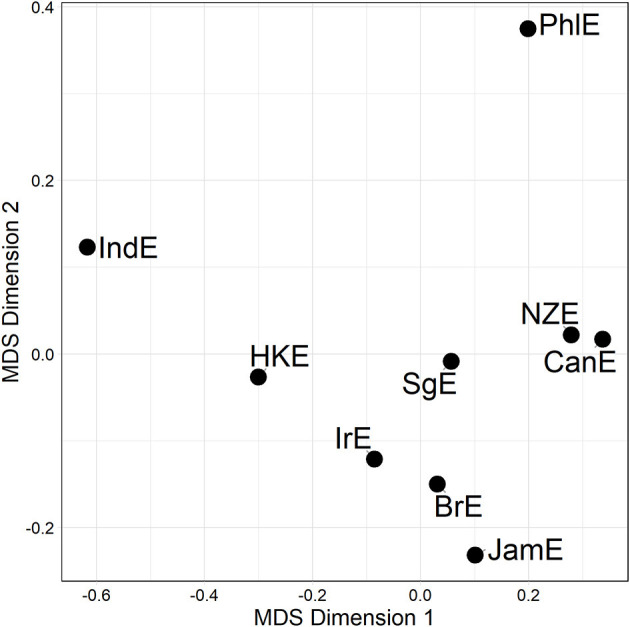
MDS representation of a compromise distance matrix merged across the genitive and particle placement alternation (all available data). Distances between data points in plot is proportional to probabilistic grammar distances between varieties.

The upshot is, then, that there is significant and substantial overlap between the genitive alternation and the particle placement alternation, while the dative alternation does not overlap with either one of the other alternations. Against this backdrop, it is instructive to combine the genitive and particle placement alternation-based distance matrices—given their overlap—without throwing the dative distance matrix into the mix. Figure 5 shows an MDS representation of this combined genitive/particle placement distance matrix.

The pattern in Figure 5 is that the Inner Circle varieties are clustered in the lower right-hand quadrant in Figure 5; this quadrant also contains JamE and SgE. PhlE and IndE are outliers. Compare this to the dative alternation-only plot (middle plot Figure 3), from which no discernible pattern arises at all.

## Discussion and Conclusion

Drawing inspiration from comparative sociolinguistics and dialectometry, we have sketched in this paper a method—Variation-Based Distance & Similarity Modeling (or VADIS for short)—that gauges the extent and structure of inter-speaker variation through assessing intra-speaker variation. VADIS specifically estimates the similarity between varieties and dialects as a function of how similar the ways are in which language users choose between different ways of saying the same thing. On the technical plane, VADIS calculates a series of multivariate models that predict speakers' and writers' linguistic choices, and utilizes three criteria to calculate similarity and distance measures: (1) Are the same constraints significant across varieties? (2) What is the extent to which constraints have similar effect strengths? (3) What is the extent to which the ranking of constraints is similar? With its focus on how people make choices and thanks to its reliance on naturalistic corpus data as data source, VADIS has a more usage-based bent than classical dialectometry, and is able to pick up differences even in cases where varieties happen to have the same inventory of forms and exhibit similar frequencies, but with possibly different underlying probabilistic grammars. We noted also that the quantitative rigor of VADIS scales up better to more varieties and more variation phenomena than classical comparative sociolinguistics.

To illustrate how VADIS can characterize (dis)similarities across and relationships between varieties, we presented a case study about three syntactic alternations (the genitive alternation, the dative alternation, and the particle placement alternation) in nine World Englishes, four of which are Inner Circle, or English-as-a-native-language, varieties (BrE, CanE, IrE, and NZE), and five of which are Outer Circle, or English-as-a-second-language, varieties (IndE, HKE, SgE, PhlE, and JamE). Key findings uncovered through VADIS may be summarized as follows.

First, we showed in section Quantification via Similarity Coefficients how VADIS can precisely quantify, via similarity coefficients, the extent to which any number of varieties are similar in terms of the probabilistic grammars that regulate any number of variables and alternations. The nine World Englishes included in our case study are overall remarkably similar to each other in terms of variation patterns: on a scale from 0 (total dissimilarity) to 1 (total similarity), core grammar scores range between Γ = 0.7 and Γ = 0.8, which is another way of saying that there is overall strong overlap with regard to the probabilistic grammars regulating variation. In other words, we are dealing with a rather solid “common core” (Quirk et al., 1985, p. 33) of the grammar of English. However, all grammatical alternations are not equal: we saw that the genitive alternation tends to be more stable across varieties than the other alternations. We interpret this as indicating that the alternations under study are differentially sensitive to “probabilistic indigenization,” which Szmrecsanyi et al. (2016, p. 133) define as “as the process whereby stochastic patterns of internal linguistic variation are reshaped by shifting usage frequencies in speakers of post-colonial varieties.” Szmrecsanyi et al. (2016, p. 133) further speculate that “the more tightly associated a given syntactic alternation is with concrete instantiations involving specific lexical items […] the more likely it is to exhibit cross-varietal indigenization effects.” Note now that the genitive alternation is an almost entirely abstract alternation without lexical anchors, unlike the dative and—in particular—the particle placement alternation.

Experimentation with subsets of the datasets further showed that spoken language production tends to be more heterogeneous and regionally unstable than written language production (that is, similarity coefficients are lower when attention is restricted to spoken materials). This may be surprising to all those who would like to emphasize that the production of spoken language is subject to processing and production constraints and biases (Hawkins, 1994; MacDonald, 2013) in a way that the production of written language is probably not. But then again, it is a well-known fact that while especially vernacular speech is “the style in which the minimum attention is given to the monitoring of speech” (Labov, 1972, p. 208), written language is more “governed by prescription” (D'Arcy and Tagliamonte, 2015, p. 255), a fact that may level out regional differences. We also saw that Inner Circle varieties form a tighter typological cluster (i.e., similarity coefficients are higher) than the Outer Circle varieties, where similarity coefficients are lower. We speculate that the comparative heterogeneity of Outer Circle varieties is likely due to substrate and contact influences, which play a more important role in the Outer Circle than in the Inner Circle.

**Figure 6:**
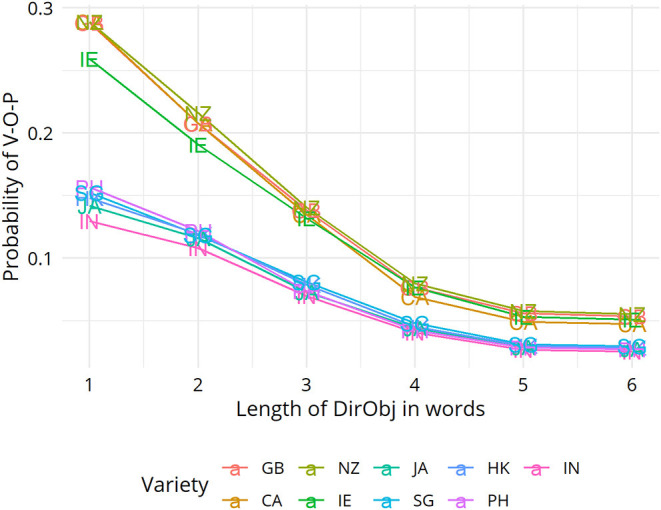
Random forest partial dependence plots of the interaction of variety with direct object length.

In section Mapping Out (dis)Similarity Relationships Between Varieties we moved on to show how the VADIS method can be used to “map out,” as it were, relationships between varieties, using techniques and visualization methods (in this case Multidimensional Scaling) widely used in dialectometry and quantitative typology. For the dative alternation, no clear picture emerged, but the plots for the genitive alternation and the particle placement alternation indicated that the Inner Circle varieties tend to cluster together. This is a pattern that has also been reported in the dialect-typological literature based on the aggregate analysis of survey data (see, e.g., Szmrecsanyi and Kortmann, 2009; Figure 2). Let us discuss the underlying variation patterns that VADIS is picking up here in more detail. As far as the genitive alternation is concerned, we know, for instance, that Inner Circle users are more sensitive to the *s*-genitive-favoring effect of possessor animacy than Outer Circle users (Heller et al., 2017, p. 18). In regard to the particle placement alternation, the dataset analyzed in Grafmiller and Szmrecsanyi (2018); Grafmiller (2018) shows that users of Inner Circle varieties are more sensitive to the length of the direct object than users of Outer Circle varieties. Consider Figure 6, which shows how across all varieties under study, the probability of the split variant (as in *I looked the word up*) decreases as the length of the direct object increases. This is the expected relationship as per the principle of end weight (Behaghel, 1909; Arnold et al., 2000). Note however how the relationship is weaker for the Outer Circle varieties (blueish lines) than for the Inner Circle varieties (yellowish lines). In other words, the principle of end weight is a more potent probabilistic predictor in Inner Circle varieties than in Outer Circle varieties. It is precisely probabilistic contrasts like these that VADIS is designed to be sensitive to.

Next we explored in section Assessing Coherence the extent to which there is coherence between (a) different lines of evidence and (b) between alternations. As to coherence between the different lines of evidence, our data suggest that there tends to be overlap between the 1st line of evidence (constraint significance) and the 2nd line of evidence (effect size), as well as between the 2nd line of evidence and the 3rd line of evidence (constraint ranking). This is true for the genitive alternation and the particle placement alternation; the distance matrices generated on the basis of data from the dative alternation do not overlap at all. As to coherence between alternations, here again the dative alternation is an outlier: the distance matrices derived from the genitive and particle placement alternations do overlap substantially, but the dative alternation distance matrix does not overlap with any of the other distance matrices. The deeper theoretical question that we are addressing here is whether grammar (or the variable parts of grammar) is essentially a collection of independent and/or independently conditioned alternations, or whether alternations actually “agree,” as it were, about differences between varieties. Our analysis suggest that we are dealing with a mixed picture. It is unexpected that and unclear why the dative alternation does not pattern with the other alternations: all three alternations are, after all, syntactic/positional alternations that are constrained by similar factors (constituent length, animacy, and so on). Further work is needed to elucidate why the dative alternation is different from the other alternations. It may be worth considering in this connection Guy (2013), a study that investigates if people consistently use stigmatized or prestige variants. Guy finds that it is not easy to demonstrate correlations in the behavior of variables, even if they are generally thought to vary along the same social dimension. The methodology in Guy (2013) is not quite comparable to ours, and he is primarily interested in social variation, not regional variation; but still, the tenor of this work is fully relevant:

every speech community has many sociolinguistic variables, do the multiple variables cohere in forming sociolects? Thus if each variable has a variant considered ‘working class', do working class speakers use all such variants simultaneously? Lectal coherence would imply that variables are correlated; if they are not, the cognitive and social reality of the “sociolect” is problematic (p. 63).

Against this backdrop, the fact that alternations do not cohere perfectly calls into question maybe not so much the reality of World Englishes but conceptions of grammar that consider grammar the aggregation of binary alternations.

One limitation of the VADIS method is that it has many free parameters—in terms of, e.g., the number of constraints to be included in the analysis, regression model structure (random intercepts, slopes, the number of constraints), methods to calculate distance matrices, and so on. This paper has suggested a number of reasonable default parameter settings to address this issue. However, we stress that decisions regarding model parameters, e.g., random effects structure, interactions, and non-linear terms in regression models or the number of trees in the random forests, are best left to individual researchers to determine based on the theoretical questions of interest, as well as the size and composition of their particular datasets. Given the risks of compounding potential problems across multiple models, careful consideration of appropriate model structures and (hyper)parameters is therefore a crucial first step in the analysis. But this step is one that must be evaluated on a case-by-case basis.

Additionally, it is worth reiterating that the validity and reliability of the VADIS method depends upon the quality and representativeness of the data sources. The present study compares standard national varieties at the most general level, and we chose the best available corpora (ICE and GloWbE) for this task. But these sources are not without their issues. Despite the best efforts of ICE compilation teams, social and demographic information is not available for some speakers, and the sampling, and hence representativeness, of some registers in each component will vary somewhat depending on the availability of English texts/speakers in a given region. GloWbE, a massive, aggregate corpus of online texts from around the world, has also been criticized for the unknown degree of variability and heterogeneity in its data sources (see e.g., Davies and Fuchs, 2015 and responses in the same issue). We therefore add a word of caution about generalizing too far beyond the present study, and stress the need for more focused comparisons of individual registers and/or regions.

On a related note, a further aspect that needs to be addressed in future work is external validation of the VADIS methodology. This paper has presented just a first case study showcasing the method and its potential, but comparing the outcome of VADIS to other types of data will be primordial to fully assess the method's strengths and limitations. We are currently exploring ways to use experimental data on speaker intuitions about the three alternations studied in this paper to provide a first step toward external validation of VADIS. Another way to externally validate the outcome of VADIS would be to use correlation analysis to determine how well the distance matrices obtained in VADIS' three lines of evidence align with distance matrices derived from other data on the alternations under study. An example of how this can be done in future work can be found in Röthlisberger (2018) who compares distance matrices derived from probabilistic models to distance matrices calculated based on morphosyntactic information found in the *Electronic World Atlas of Varieties of English* (Kortmann and Lunkenheimer, 2013).

And this takes us to directions for future research, which include the following. The case study presented here is obviously just a first step, and the similarity coefficients and core grammar scores we presented need comparative contextualization. In the realm of English linguistics, we need to include more or different alternations (including phonological, morphological, and function word alternations), and the analysis needs to be extended to more or different regional varieties of English. Beyond English linguistics, we need comparative analysis covering other languages: how stable or unstable are the probabilistic grammars of varieties of e.g., Spanish or French compared to varieties of English? Do we see the same sort of split between native and non-native varieties? And so on. Last but not least, VADIS can be adapted to study not geographical varieties (as we did here) but historical and situational varieties. VADIS could then be used to measure probabilistic stability across time and registers. Recent work in this respect is quite promising. Grafmiller (2018), for example, adopts a VADIS-like approach to investigate stylistic variation in English genitives, and finds that the methods yield patterns in accordance with previous work on register variation. He shows that genitive use in press writing, though still quite distinct from spoken genitives, nevertheless became increasingly more informal/colloquial (e.g., Jucker, 1993) over the twentieth century. Over the same time period, genitives in academic writing also changed dramatically, albeit in ways that do not track with typical colloquialization trends (see e.g., Biber and Gray, 2016; Hyland and Jiang, 2017).

## Data Availability Statement

Publicly available datasets were analyzed in this study. This data can be found here: https://osf.io/3gfqn/.

## Author Contributions

BS, JG, and LR collaborated on the conception and design of the study. Data was collected and prepared by BS and JG. A first draft of the paper was written by BS. JG and LR wrote sections of the paper. BS took care of the final and submitted version of the manuscript which was read, revised, and approved by LR and JG.

### Conflict of Interest

The authors declare that the research was conducted in the absence of any commercial or financial relationships that could be construed as a potential conflict of interest.

## Footnotes

^1^The concept of a probabilistic grammar thus largely overlaps with what variationist sociolinguists refer to as a “variable grammar,” defined by Tagliamonte (2006, p. 240), citing Poplack and Tagliamonte (2001, p. 91), as being represented by “the hierarchy of constraints constituting each factor [that regulates variation]”.

^2^http://ice-corpora.net/ice/index.html

^3^https://www.english-corpora.org/glowbe/

^4^We experimented with predictor sets of different sizes, from *p* = 5 to *p* = 10. In principle, larger predictor sets are preferable to smaller predictor sets, but then again including too many predictors that turn out as insignificant in many cases is problematic. Given these principles *p* = 8 seemed like a good compromise for the case study we report here. See Tamaredo et al. (2019) for a VADIS analysis that uses *p* = 5.

^5^The method as outlined here does not distinguish between different types of constraints, e.g., between what Tamminga et al. (2016, p. 303) term sociostylistic factors (*s*-conditioning), internal linguistic factors (*i*-conditioning), and physiological and psycholinguistic factors (*p*-conditioning). Note however that the method can be easily adapted to restrict attention to only particular types of constraints.

^6^The concordance statistic (or index) represents the probability that the model will rank any randomly chosen observation of the predicted variant higher than any randomly chosen observation of the alternate variant. C is equal to the area under the receiver operating characteristic curve. Note that model fit only matters for VADIS to the extent that the model fits are acceptable and reasonably close to one another across the same alternation. One probably should not compare models with C values of 0.75 and 0.95, but a range of 0.02 or 0.03 seems perfectly reasonable.

^7^We acknowledge that this step relies on null hypothesis significance testing based on ultimately arbitrary alpha levels, which is increasingly controversial. Note, however, that VADIS also includes two other lines of evidence which are more nuanced. The main reason why we include step 3 is that checking significances is a customary line of evidence in classical comparative sociolinguistics, and so for the sake of continuity with the extant literature VADIS also considers this criterion.

^8^A Bonferroni correction could in principle be used to make the alpha level more conservative, but we refrain from doing so here since our main interest lies with comparative analysis (using significance as an auxiliary criterion), and not with statistical significance *per se*.

^9^A disadvantage of including all estimates in the model, also the ones of constraints that do not reach significance, is that the latter may not be very reliable. However, we have opted not to use significance as an arbitrary cut-off point in this line of evidence in order not to repeat the weakness of the first line (see also footnote 7 in that respect).

^10^The number of trees in the forests was set to 500, and the number of predictors sampled (“mtry”) was set to 3. All other hyperparameters were left at the default values for the package functions.

^11^Note that no random effects were included given that mixed effects random forests are not yet fully implemented for classification problems.

^12^We stress that this measurement is only about the ranking of the constraints, and does not take graded differences in terms of the actual variable importance scores into account (see Strobl et al., 2009, p. 336 on why variable importance scores should not be directly compared across models). Graded differences are anyway covered by the 2^nd^ line of evidence (step 4).

^13^The distances that are calculated in VADIS are transitive, thus if the distance between variety A and B is 0, and the distance between B and C is 0, then the distance between variety A and C will also be 0.

^14^In this study, we are using R's cmdscale() function to obtain MDS solutions.

^15^We use the fuse() function in R package analogue to fuse distance matrices (see https://cran.r-project.org/web/packages/analogue/analogue.pdf). All input matrices are weighted equally. This could in principle be changed, but we see no compelling reason to weigh up or down particular lines of evidence.

^16^We use the mantel() function in R package vegan to calculate Mantel coefficients (see https://cran.r-project.org/web/packages/vegan/vegan.pdf).
